# Multimodal radiomics based on dynamic contrast-enhanced ultrasound for predicting cervical lymph node metastasis in papillary thyroid carcinoma with Hashimoto’s thyroiditis

**DOI:** 10.3389/fonc.2026.1840185

**Published:** 2026-10-14

**Authors:** Kairen Zhang, Dan Zhao, Gechao Wang, Yang Gu, Xiaofeng Wu, Fenglin Dong

**Affiliations:** Department of Ultrasound, The First Affiliated Hospital of Soochow University, Suzhou, China

**Keywords:** cervical lymph node metastasis, contrast-enhanced ultrasound, Hashimoto’s thyroiditis, papillary thyroid carcinoma, radiomics

## Abstract

**Objective:**

This study aimed to develop and validate a multimodal predictive model by integrating radiomic features from B-mode ultrasound (BMUS) and dynamic contrast-enhanced ultrasound (DCE-US) parametric imaging, as well as clinical and conventional ultrasound features, for preoperative prediction of cervical lymph node metastasis (CLNM) in patients with Hashimoto’s thyroiditis (HT) complicated with papillary thyroid carcinoma (PTC).

**Methods:**

A total of 191 patients with pathologically confirmed HT-complicated PTC were retrospectively enrolled between December 2023 and July 2025. Patients were randomly divided into a training set (n=133) and a validation set (n=58) at a 7:3 ratio using a random number generator. Radiomic features were extracted from BMUS images and DCE-US peak enhancement parametric maps. Feature stability was assessed using intraclass and interclass correlation coefficients (ICC). Key features were selected through a sequential process including the Mann–Whitney U test, recursive feature elimination (RFE), and least absolute shrinkage and selection operator (LASSO) regression, followed by calculation of the radiomics score (Rad-score). Independent predictors of CLNM were identified using multivariate logistic regression incorporating clinical and ultrasound features. Three predictive models were constructed: a clinical-ultrasound model, a radiomics model, and a combined multi-modal model. Model performance was evaluated using receiver operating characteristic (ROC) curves, calibration curves, decision curve analysis (DCA), net reclassification improvement (NRI), and integrated discrimination improvement (IDI). Model interpretability was enhanced using Shapley Additive Explanations (SHAP) analysis.

**Results:**

TG-Ab, TR-Ab, extrathyroidal extension, filling defects, BMUS Rad-score, and DCE-US Rad-score were independent predictors of CLNM (P < 0.05). In the training and validation sets, the combined model achieved area under the curve (AUC) values of 0.936 and 0.916, respectively, which were significantly higher than those of the radiomics model (AUC = 0.825, 0.876) and the clinical-ultrasound model (AUC = 0.760, 0.800; DeLong test, P < 0.05). Calibration curves showed good model fitting. DCA confirmed that the combined model provided greater clinical net benefit. NRI and IDI verified that the combined model significantly improved CLNM prediction performance. SHAP analysis revealed that DCE-US Rad-score was the most important predictor for CLNM.

**Conclusion:**

A multi-modal predictive model combining dual-modality ultrasound radiomic scores (BMUS and DCE-US) with clinical and ultrasound features can effectively and robustly predict CLNM in patients with HT-complicated PTC and performs better than single-modal models. This model provides an objective reference for the formulation of individualized surgical strategies.

## 1 Introduction

The accurate preoperative prediction of cervical lymph node metastasis (CLNM) is of paramount importance for optimizing surgical strategies in patients with papillary thyroid carcinoma (PTC). This is particularly relevant in the subgroup of patients with concurrent Hashimoto’s thyroiditis (HT), a common autoimmune thyroid disorder that not only complicates the sonographic evaluation but may also influence tumor biology and metastatic behavior (1, 2). Although the overall incidence of CLNM in HT‐PTC patients is relatively lower than in PTC patients without HT, a tendency toward extensive prophylactic lymph node dissection persists in clinical practice. This exposes patients to increased surgical risks—including recurrent laryngeal nerve injury, hypoparathyroidism, and cervical morbidity—without consistently demonstrated oncologic benefit (1, 2). Moreover, the diffuse parenchymal changes and heterogeneous echotexture induced by HT substantially increase the diagnostic difficulty of conventional ultrasound, often obscuring the primary tumor margins and reducing the accuracy of preoperative assessment. These clinical challenges highlight an urgent need for more precise and reliable non‐invasive tools to stratify CLNM risk in this specific population.

Conventional ultrasound, while widely used as the first‐line imaging modality for thyroid evaluation, suffers from inherent limitations including operator dependency, subjective interpretation, and reliance on macroscopic morphological features (3). Dynamic contrast‐enhanced ultrasound (DCE‐US) has emerged as a valuable adjunct to overcome some of these constraints. By enabling real‐time visualization of microvascular perfusion, DCE‐US provides quantitative hemodynamic parameters that reflect tumor angiogenesis and have been associated with aggressive tumor behavior and metastatic potential (4). However, the visual interpretation of DCE‐US images remains largely qualitative and experience‐dependent, limiting its full diagnostic potential.

The advent of radiomics has revolutionized medical image analysis by converting standard medical images into high‐dimensional, mineable data. Through the extraction of numerous quantitative features—including first‐order statistics, texture, and wavelet‐based characteristics—radiomics can capture subtle intratumoral heterogeneity and phenotypic patterns that are imperceptible to the human eye (5). This approach addresses the fundamental limitation of inadequate feature extraction in conventional imaging and provides a more objective and reproducible framework for tumor characterization. In the context of thyroid cancer, recent studies have explored the application of ultrasound‐based radiomics for predicting CLNM. Liu et al. developed a multimodal ultrasound radiomics model integrating conventional ultrasound, CEUS, and elastography, achieving promising predictive performance (6). Similarly, Wei et al. constructed a CEUS‐based radiomics nomogram with favorable results in preoperative CLNM risk stratification (7). Despite these advances, the majority of existing models have been developed in general PTC cohorts without specific focus on HT‐complicated patients, and few have incorporated DCE‐US parametric imaging features to capture dynamic perfusion information.

This study aimed to develop and evaluate a multimodal radiomics model integrating BMUS and DCE-US parametric imaging with clinical and serological markers for prediction of CLNM in patients with PTC and HT, and to assess its potential for refining risk stratification and guiding surgical planning.l

## 2 Materials and methodology

### 2.1 Methods

This retrospective study, conducted between December 2023 and July 2025, enrolled consecutive patients with malignant thyroid nodules. The study was performed in full compliance with the Declaration of Helsinki and was approved by the Institutional Review Board (IRB) of The First Affiliated Hospital of Soochow University (approval No. 2026279). All patients had already provided written informed consent for the administration of SonoVue^®^ contrast agent prior to the DCE-US examination, as this procedure is part of routine clinical care. For the subsequent retrospective analysis of anonymized imaging and clinical data, the IRB granted a formal waiver of additional informed consent, based on the following justifications: (1) the research involved no more than minimal risk to subjects; (2) it did not alter any clinical management decisions; and (3) all data were fully anonymized prior to analysis to guarantee patient confidentiality. The inclusion criteria were as follows: (1) postoperative pathologically confirmed unifocal papillary thyroid carcinoma; (2) coexisting Hashimoto’s thyroiditis; (3) complete and diagnostically adequate thyroid ultrasound images; and (4) comprehensive clinical data including demographic information, ultrasound features, and thyroid-related immune markers (TPO-Ab, TG-Ab, and TR-Ab). Patients were excluded if they had poor-quality ultrasound images, received preoperative interventional therapy such as ablation, or had incomplete clinical data. A total of 191 eligible patients were finally included and randomly divided into a training cohort (n=133) and a validation cohort (n=58) at a 7:3 ratio (**Supplementary Figure 1**).

### 2.2 Image acquisition and analysis

In this study, a Mindray Resona 7 or Resona 9 ultrasound system was used, equipped with high-frequency linear array transducers L11-3 (3–11 MHz) and L14-5 (5–14 MHz), respectively. All participants were examined in the supine position with both arms abducted to fully expose the thyroid region. Conventional BMUS was first performed to document the largest cross-section of each lesion. Subsequently, 1.2–2.4 mL of sulfur hexafluoride microbubble contrast agent (SonoVue^®^; Bracco, Italy) was administered as a bolus injection at a rate of 1.5 mL/s, followed by a 5-mL saline flush. Simultaneously with contrast injection, a timer and dynamic storage function were initiated. The transducer was then held stationary at the largest cross-section of the lesion for 2 minutes to capture the full contrast-enhanced ultrasound sequence at a constant frame rate of 15 Hz. All CEUS images were archived as dynamic image series in DICOM format.

Two sonographers with more than 15 years of experience in thyroid ultrasound retrospectively evaluated ultrasound features while blinded to clinical information. Any discrepancies in the assessment of grayscale and contrast-enhanced ultrasound features between the two readers were resolved by a third senior sonographer until a consensus was reached. Features evaluated on BMUS included: lesion size (maximum diameter measured by ultrasound), echogenicity (isoechoic, hyperechoic, hypoechoic, or markedly hypoechoic), anteroposterior/transverse diameter ratio >1 (yes/no), margin (well-defined, obscured, or irregular), extrathyroidal extension (ETE) (yes/no), and microcalcifications (absent or present).Features evaluated on contrast-enhanced ultrasound included: enhancement pattern (Scattered centripetal, or centrifugal), enhancement homogeneity (homogeneous or heterogeneous), and filling defect (absent or present).The cutoff values for thyroid function-related antibodies were based on routine clinical reference values from the Guidelines for the Diagnosis and Treatment of Thyroid Diseases (2023 Edition): TG-Ab ≤ 60 IU/mL, TR-Ab ≤ 1.75 IU/L, and TPO-Ab ≤ 34 IU/mL.

### 2.3 Radiomics analysis

#### 2.3.1 BMUS and DCE-US image segmentation

For BMUS images, a sonographer with > 5 years of clinical experience manually delineated the region of interest (ROI) along the nodule contour on the maximum cross-sectional image using the 3D Slicer software. For thyroid nodule (TN) segmentation on DCE-US reconstructed images, the CEUS video was first processed using offline external perfusion analysis software (TIC tool) to generate DCE-US reconstruction images based on peak enhancement (PE), a key perfusion parameter. The time–intensity curve (TIC) analysis derived from DCE-US provides quantitative parameters that reflect the dynamic perfusion process of the lesion. Among these, PE represents the maximum signal intensity achieved during contrast agent accumulation and has been demonstrated to be closely associated with tumor aggressiveness and the risk of lymph node metastasis (3, 8, 9). Utilizing pixel-wise PE values, a parametric map reflecting the microvascular perfusion intensity of thyroid nodules was generated. In this map, the luminance value of each pixel corresponds to the maximum enhancement intensity at that location throughout the imaging process, thereby enabling visualization of the spatial distribution of intratumoral microvessels. The nodule ROI was subsequently segmented on both BMUS and DCE-US reconstructed images.

#### 2.3.2 Radiomics feature extraction and stability evaluation

For radiomics feature extraction, we employed the open-source software 3D Slicer (version 5.7.0). Prior to feature extraction, all images underwent standardized preprocessing, including resampling to isotropic voxels of 1 mm × 1 mm × 1 mm, grayscale discretization with a bin width of 25, and wavelet transformation. A total of 837 radiomics features were extracted from each region of interest (ROI), including first-order statistics, shape-based features, and texture features derived from the gray-level co-occurrence matrix (GLCM), gray-level run-length matrix (GLRLM), gray-level size-zone matrix (GLSZM), neighboring gray-tone difference matrix (NGTDM), and gray-level dependence matrix (GLDM). All features were extracted from the original and wavelet-transformed images.

To assess the reliability of radiomic feature extraction, the intraclass and interclass correlation coefficients (ICC) were calculated. For this analysis, 30 ultrasound images were randomly selected; the same physician re-delineated regions of interest (ROIs) after an interval of one month to evaluate intra-observer reproducibility, while a second senior physician independently delineated ROIs on the same set of images to assess inter-observer reproducibility. Only features with ICC values greater than 0.75 were regarded as having satisfactory consistency and were retained for subsequent analyses.

#### 2.3.3 Radiomics feature selection and model construction

In this study, ultrasound imaging features derived from BMUS and DCE-US were systematically selected and optimized. First, the Z-score standardization algorithm was applied to preprocess the features in the training cohort, eliminating the influence of differing numerical ranges on the analysis results and improving model convergence and stability. Subsequently, an initial selection of the extracted features was performed using the Mann–Whitney U test, retaining only those features with statistically significant differences (P < 0.05).

Recursive feature elimination (RFE) is a classical feature-selection method based on a greedy recursive elimination strategy. In this study, RFE was combined with a random forest classifier to robustly evaluate feature importance through five-fold cross-validation. In each cross-validation round, the least important features were recursively eliminated, ultimately retaining the top 10% of the features from the candidate cohort obtained after initial selection. This process achieved preliminary feature reduction.

Finally, based on the feature cohort obtained from the previous steps, the least absolute shrinkage and selection operator (LASSO) regression model was used to identify key features with significant predictive values. LASSO regression was selected as the dimensionality reduction and feature selection method for the following reasons: First, radiomics data are typically high-dimensional, with substantial multicollinearity among features. LASSO applies L1-penalization that performs continuous shrinkage and automatic feature selection simultaneously, effectively reducing the overfitting risk while maintaining model interpretability, which is a critical advantage given our relatively limited sample size. Second, the primary goal of this study was to construct a clinically practical and parsimonious predictive model. LASSO yields a sparse solution, retaining only the most informative features, enabling a straightforward Rad-score calculation that can be readily implemented in clinical settings. This approach has been extensively validated and recommended as the preferred dimensionality reduction method in radiomics studies aimed at developing clinically deployable risk stratification tools.

The model was implemented using Python’s Scikit-learn toolkit, and the optimal penalty coefficient λ was determined via 10-fold cross-validation. The BMUS Rad-score and DCE-US Rad-score were then calculated as linear combinations of the selected features weighted by their respective LASSO coefficients. Specifically, the Rad-score was computed using the following formula:Rad-score = β_0_ + Σ(β_i_ × X_i_), where X_i_ represents the standardized value of each selected radiomics feature, and β_i_ is the corresponding LASSO regression coefficient. The intercept (β_0_) and the coefficients for all selected features are provided in **Supplementary Table S1**. **Figure 1** illustrates the overall workflow of this study. This study was reported in accordance with the Checklist for Artificial Intelligence in Medical Imaging (CLAIM). The completed CLAIM checklist is provided as **Supplementary Table S2**.

**Figure 1:**
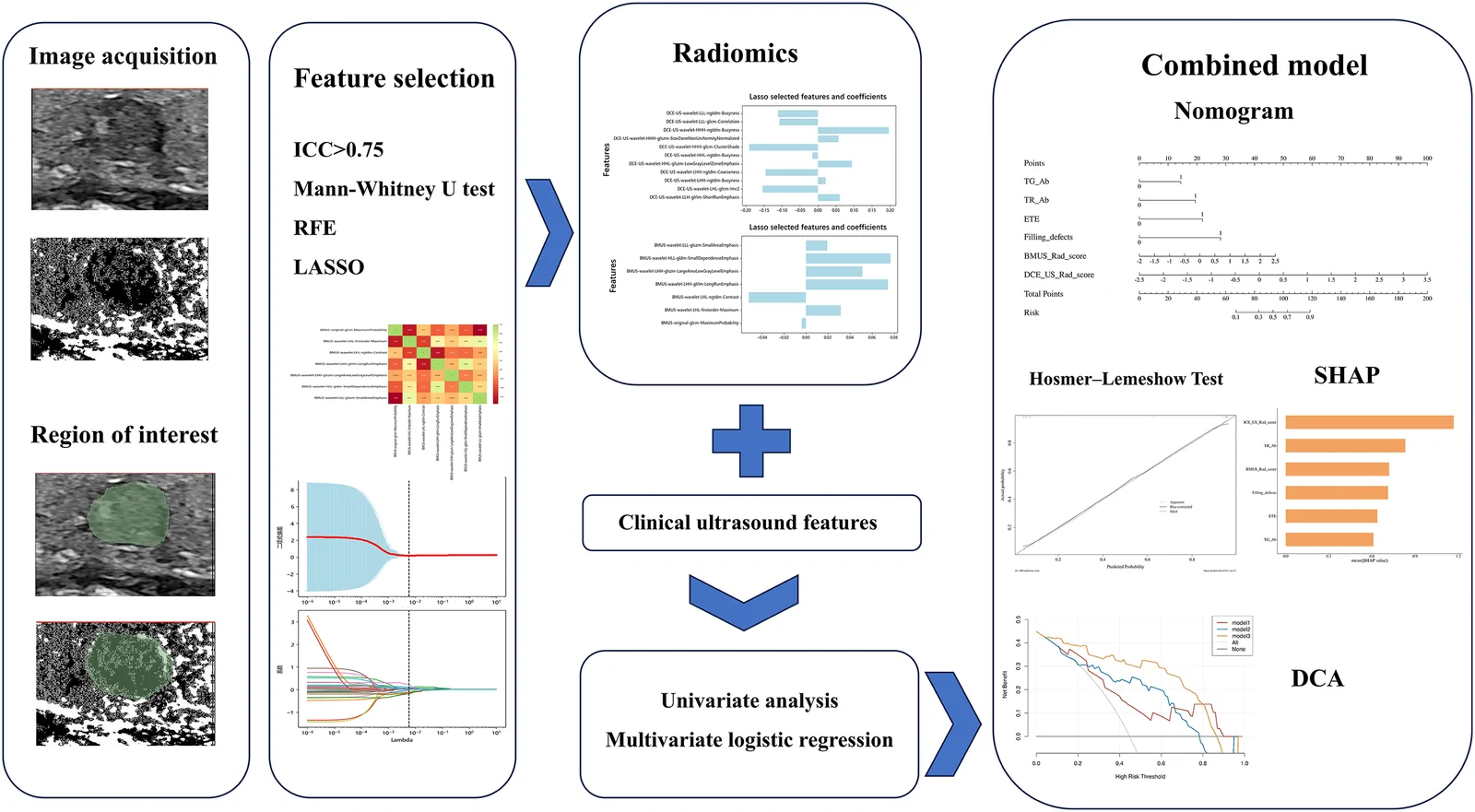
Flow chart of ultrasound imaging. ICC, Intra- and interclass correlation coefficient; RFE, Recursive Feature Elimination; LASSO, Least Absolute Shrinkage and Selection Operator; SHAP, SHapley Additive exPlanations; DCA, Decision Curve Analysis.

#### 2.3.4 Internal cross-validation for radiomics model stability

To evaluate the stability of the LASSO-based radiomics feature selection, we performed 100 repetitions of 10-fold stratified cross-validation within the training cohort, with different random seeds for each repetition. In each fold, the LASSO model was trained on the training subset and evaluated on the validation subset, and the AUC was recorded. This process yielded 1,000 AUC values for the BMUS model and 1,000 for the DCE-US model. The mean AUC, standard deviation (SD), and standard error (SE) were calculated for each model to assess discriminative performance and estimation stability.

### 2.4 Construction and evaluation of Combined model and nomogram

Initially, a univariate analysis was conducted to select clinical and ultrasound features, as well as radiomics scores, to identify potential predictors of CLNM. Subsequently, a multivariate logistic regression analysis was performed to pinpoint independent risk factors associated with CLNM, with statistical significance at P < 0.05. For each factor, the odds ratio (OR) and corresponding 95% confidence interval (95% CI) were calculated. Based on the multivariate analysis results, a predictive model for CLNM in patients with HT and PTC was developed. The model’s calibration performance was assessed using calibration curves in conjunction with the Hosmer–Lemeshow goodness-of-fit test. Its discriminative ability was evaluated by calculating the area under the curve (AUC) in both the training and validation cohorts. To further evaluate clinical utility, decision curve analysis (DCA) was employed to assess the net benefit of the combined nomogram. A comprehensive assessment of the model’s diagnostic performance, calibration, and clinical applicability was conducted based on the AUC, calibration curves, and DCA results. To compare the predictive performance of different models, the DeLong test was used to statistically evaluate differences in AUC values. Additionally, net reclassification improvement (NRI) and integrated discrimination improvement (IDI) were calculated to quantify the enhancement in predictive capacity of the combined ultrasound radiomics model relative to the radiomics-only model and the clinical ultrasound model. IDI reflects the overall improvement in predicted probability, specifically the increase in average sensitivity minus any loss in average specificity. A positive IDI value (IDI > 0) indicates improved predictive performance, IDI < 0 suggests a decline, and IDI = 0 implies no significant improvement. To enhance the interpretability of the final combined model, Shapley additive explanations (SHAP) analysis was applied. Based on cooperative game theory, this method quantitatively evaluates the contribution and direction of influence of each predictive feature on the model output, thereby identifying and visualizing the core features driving model decisions. This approach clarifies the positive or negative associations between each feature and the predicted outcome, providing an intuitive and credible explanation for the model’s decision-making process. To further evaluate the model’s predictive performance for central lymph node metastasis, we defined a subgroup of patients with central lymph node metastasis within the validation cohort. Patients in the validation cohort were stratified based on lymph node metastasis status: those with lateral cervical lymph node metastasis were excluded, and only patients with no lymph node metastasis or isolated central lymph node metastasis were retained. The original partitioning of the training and validation cohorts remained unchanged. The combined model was used to compute the predicted probabilities for all eligible patients in this restricted validation subgroup, and the predictive performance of the model was then evaluated.

### 2.5 Statistical analysis

All statistical analyses were performed using R (version 3.6.1), IBM SPSS Statistics (version 27.0), and MedCalc (version 20.027). Continuous variables were first tested for normality using the Shapiro–Wilk test. Normally distributed variables are expressed as mean ± standard deviation (SD), while non-normally distributed variables are reported as median with interquartile range (IQR). Intergroup comparisons for continuous variables were performed using the independent samples t-test for normally distributed data and the Mann–Whitney U test for non-normally distributed data. Categorical variables were compared using the chi-square test or Fisher’s exact test, as appropriate. To eliminate the influence of dimensional differences, the original radiomics scores were standardized to a mean of 0 and standard deviation of 1 (Z-score normalization) prior to model development. All statistical tests were two-sided, and a P-value < 0.05 was considered statistically significant.

## 3 Results

### 3.1 Clinical data

A total of 191 patients (mean age, 43.42 years) were included in this study, including 86 patients with CLNM and 105 without CLNM. All 191 patients underwent thyroidectomy with central lymph node dissection. All resected lymph nodes were pathologically examined. The training cohort included 60 patients with CLNM and 73 patients without CLNM, and the validation cohort included 26 patients with CLNM and 32 patients without CLNM. There were no statistical differences in the baseline characteristics between the training and validation cohorts (**Table 1**).

**Table 1:** Baseline characteristics of patients in the training and validation cohort.

Variable	Overall population	Training cohort	Validation cohort	P-value
Clinical features	N= 191	N = 133	N = 58	
Age, Mean ± SD	43.42 ± 12.65	43.46 ± 13.13	43.33 ± 11.58	0.948
Gender, n(%)				0.540
Male	44 (23.04)	29 (21.80)	15 (25.86)	
Female	147 (76.96)	104 (78.20)	43 (74.14)	
Location, n(%)				0.551
Right lobe	94 (49.21)	62 (46.62)	32 (55.17)	
Left lobe	79 (41.36)	58 (43.61)	21 (36.21)	
Isthmus	18 (9.42)	13 (9.77)	5 (8.62)	
Tumor size, mean ± SD	13.13 ± 9.53	12.99 ± 9.54	13.43 ± 9.60	0.771
TPO-Ab, n(%)				0.472
≤34	88 (46.07)	59 (44.36)	29 (50.00)	
>34	103 (53.93)	74 (55.64)	29 (50.00)	
TG-Ab, n(%)				0.854
≤60	87 (45.55)	60 (45.11)	27 (46.55)	
>60	104 (54.45)	73 (54.89)	31 (53.45)	
TR-Ab, n(%)				0.39
≤1.75	103 (53.93)	69 (51.88)	34 (58.62)	
>1.75	88 (46.07)	64 (48.12)	24 (41.38)	
Cervical lymph node metastasis, n(%)				0.971
No	105 (54.97)	73 (54.89)	32 (55.17)	
Yes	86 (45.03)	60 (45.11)	26 (44.83)	
Ultrasound features				
Echogenicity, n(%)				1.000
Isoechoic	9 (4.71)	6 (4.51)	3 (5.17)	
Hypoechoic and Markedly hypoechoic	169 (88.48)	118 (88.72)	51 (87.93)	
Hyperechoic	13 (6.81)	9 (6.77)	4 (6.90)	
Composition, n(%)				0.754
Cystic or almost completely cystic	7 (3.66)	4 (3.01)	3 (5.17)	
Solid or almost completely solid	184 (96.34)	129 (96.99)	55 (94.83)	
Margin, n(%)				0.333
Smooth	69 (36.13)	51 (38.35)	18 (31.03)	
Blurred or irregular	122 (63.87)	82 (61.65)	40 (68.97)	
Shape, n(%)				0.729
Wider-than-tall	76 (39.79)	54 (40.60)	22 (37.93)	
Taller-than-wide	115 (60.21)	79 (59.40)	36 (62.07)	
ETE, n (%)				0.723
No	148 (77.49)	104 (78.20)	44 (75.86)	
Yes	43 (22.51)	29 (21.80)	14 (24.14)	
Microcalcification, n(%)				0.755
No	69 (36.13)	49 (36.84)	20 (34.48)	
Yes	122 (63.87)	84 (63.16)	38 (65.52)	
CDFI, n(%)				0.411
Level 0	75 (39.27)	50 (37.59)	25 (43.10)	
Level 1	59 (30.89)	45 (33.83)	14 (24.14)	
Level 2	57 (29.84)	38 (28.57)	19 (32.76)	
Enhancement type, n(%)				0.588
None or iso-enhancement	27 (14.14)	20 (15.04)	7 (12.07)	
Hypo enhancement	164 (85.86)	113 (84.96)	51 (87.93)	
Enhancement homogeneity, n(%)				0.339
Homogeneous	34 (17.80)	26 (19.55)	8 (13.79)	
Heterogeneous	157 (82.20)	107 (80.45)	50 (86.21)	
Filling defects, n(%)				0.482
Absent	154 (80.63)	109 (81.95)	45 (77.59)	
Present	37 (19.37)	24 (18.05)	13 (22.41)	
Enhancement direction, n(%)				0.672
Scattered	50 (26.18)	36 (27.07)	14 (24.14)	
Centripetal or centrifugal	141 (73.82)	97 (72.93)	44 (75.86)	
Radiomics score				
BMUS-Rad-score, Mean ± SD	0.45 ± 0.21	0.45 ± 0.20	0.44 ± 0.22	0.713
DCE-US Rad-score, Mean ± SD	0.45 ± 0.28	0.45 ± 0.28	0.44 ± 0.27	0.750

### 3.2 Radiomics features

First, 837 radiomics features were extracted from BMUS and reconstructed DCE-US images, and the stable features of ICC>0.75 were selected for the Mann–Whitney U test; the top 10% of the features were retained by RFE; the dimensionality was reduced by LASSO regression; the optimal regularization parameter λ was selected using 10-fold cross-verification; and finally, seven key features were selected by BMUS screening. DCE-US obtained 11 key features. Subsequently, a dual-modal ultrasound radiomics scoring model was constructed based on the BMUS and DCE-US radiomics scores(**Supplementary Figures 1, 2**). **Tables 1**, **2** presents specific information on the selected features. Based on the above characteristics and coefficients, the BMUS Rad-score and DCE-US Rad-score were calculated. In both the training and validation cohorts, the BMUS and DCE-US Rad- scores in the CLNM group were significantly higher than those in the NCLNM group.

**Table 2:** Univariate analysis in the training cohort.

Variable	Overall population(n= 133)	NCLNM(n= 73)	CLNM(n= 60)	P-value
Clinical features				
Age, Mean ± SD	43.46 ± 13.13	44.79 ± 13.53	41.83 ± 12.54	0.197
Gender, n(%)				0.648
Male	29 (21.80)	17 (23.29)	12 (20.00)	
Female	104 (78.20)	56 (76.71)	48 (80.00)	
Location, n(%)				0.418
Right lobe	62 (46.62)	34 (46.58)	28 (46.67)	
Left lobe	58 (43.61)	34 (46.58)	24 (40.00)	
Isthmus	13 (9.77)	5 (6.85)	8 (13.33)	
Tumor size, mean ± SD	12.99 ± 9.54	11.47 ± 9.10	14.85± 9.80	0.041
TPO-Ab, n(%)				0.627
≤34	59 (44.36)	31 (42.47)	28 (46.67)	
>34	74 (55.64)	42 (57.53)	32 (53.33)	
TG-Ab, n(%)				0.005
≤60	60 (45.11)	41 (56.16)	19 (31.67)	
>60	73 (54.89)	32 (43.84)	41 (68.33)	
TR-Ab, n(%)				<0.01
≤1.75	69 (51.88)	49 (67.12)	20 (33.33)	
>1.75	64 (48.12)	24 (32.88)	40 (66.67)	
Ultrasound features				
Echogenicity, n(%)				0.773
Isoechoic	6 (4.51)	3 (4.11)	3 (5.00)	
Hypoechoic and Markedly hypoechoic	118 (88.72)	64 (87.67)	54 (90.00)	
Hyperechoic	9 (6.77)	6 (8.22)	3 (5.00)	
Composition, n(%)				1
Cystic or almost completely cystic	4 (3.01)	2 (2.74)	2 (3.33)	
Solid or almost completely solid	129 (96.99)	71 (97.26)	58 (96.67)	
Margin, n(%)				0.722
Smooth	51 (38.35)	27 (36.99)	24 (40.00)	
Blurred or irregular	82 (61.65)	46 (63.01)	36 (60.00)	
Shape, n(%)				0.100
Wider-than-tall	54 (40.60)	25 (34.25)	29 (48.33)	
Taller-than-wide	79 (59.40)	48 (65.75)	31 (51.67)	
ETE, n (%)				<.001
No	104 (78.20)	66 (90.41)	38 (63.33)	
Yes	29 (21.80)	7 (9.59)	22 (36.67)	
Microcalcification, n(%)				0.138
No	49 (36.84)	31 (42.47)	18 (30.00)	
Yes	84 (63.16)	42 (57.53)	42 (70.00)	
CDFI, n(%)				0.496
Level 0	50 (37.59)	30 (41.10)	20 (33.33)	
Level 1	45 (33.83)	25 (34.25)	20 (33.33)	
Level 2	38 (28.57)	18 (24.66)	20 (33.33)	
Enhancement type, n(%)				0.991
None or iso-enhancement	20 (15.04)	11 (15.07)	9 (15.00)	
Hypo enhancement	113 (84.96)	62 (84.93)	51 (85.00)	
Enhancement homogeneity, n(%)				0.101
Homogeneous	26 (19.55)	18 (24.66)	8 (13.33)	
Heterogeneous	107 (80.45)	55 (75.34)	52 (86.67)	
Filling defects, n(%)				<0.001
Absent	109 (81.95)	70 (95.89)	39 (65.00)	
Present	24 (18.05)	3 (4.11)	21 (35.00)	
Enhancement direction, n(%)				0.925
Scattered	36 (27.07)	20 (27.40)	16 (26.67)	
Centripetal or centrifugal	97 (72.93)	53 (72.60)	44 (73.33)	
Radiomics score				
BMUS-Rad-score, Mean ± SD	0.02 ± 0.97	-0.43 ± 0.76	0.56 ± 0.92	<0.001
DCE-US Rad-score, Mean ± SD	0.02 ± 1.01	-0.53 ± 0.76	0.68 ± 0.85	<0.001

#### 3.2.1 Stability of radiomics feature selection

Repeated cross-validation demonstrated stable and reproducible feature selection for both radiomics models. Across 1,000 validation iterations, the BMUS model achieved a mean AUC of 0.725 (SD = 0.144, SE = 0.005), and the DCE-US model achieved a mean AUC of 0.790 (SD = 0.124, SE = 0.004). The small standard errors (≤ 0.005) confirmed that the performance estimates were highly robust and not driven by random data partitioning. A paired comparison further revealed that the DCE-US model significantly outperformed the BMUS model, with a mean AUC difference of 0.064 (95% CI: 0.053–0.076, p < 0.001). These findings confirmed the stability of our radiomics-based feature screening and modeling process.

### 3.3 Construction and verification of combined model and nomogram

In the training group, univariate analysis showed that there were statistically significant differences in TG-Ab, TR-Ab, ETE, enhancement loss, BMUS radiomics score, and DCE-US radiomics score between the metastatic and non-metastatic cohorts (P<0.05) (**Table 2**). Subsequently, multivariate logistic regression analysis was performed on the above six indicators, and the results showed that TG-Ab, TR-Ab, ETE, enhancement defects, BMUS radiomics score, and DCE-US radiomics score were independent influencing factors for CLNM in patients with HT and PTC (P<0.05) (**Table 3**). Based on these independent influencing factors, three prediction models were constructed: Model 1 (clinical-ultrasound model) included TG-Ab, TR-Ab, ETE, and enhancement defects; Model 2 (radiomics model) included BMUS and DCE-US radiomics scores; and Model 3 (combined model) contained all six independent influencing factors.

**Table 3:** Multivariate logistic regression results for combined model.

Variable	β	S.E	Z	P-value	OR (95%CI)
Intercept	-2.58	0.64	-4.03	<0.001	0.08 (0.02 ~ 0.27)
TG-Ab, n(%)					
≤60					1.00 (Reference)
>60	1.23	0.59	2.08	0.037	3.43 (1.08 ~ 10.92)
TR-Ab, n(%)					
≤1.75					1.00 (Reference)
>1.75	1.67	0.59	2.82	0.005	5.29 (1.66 ~ 16.82)
Enhancement defects					
No					1.00 (Reference)
Yes	2.41	0.83	2.91	0.004	11.11 (2.20 ~ 56.24)
ETE					
No					1.00 (Reference)
Yes	1.87	0.71	2.63	0.009	6.49 (1.61 ~ 26.20)
BMUS-Rad-score	0.9	0.36	2.5	0.013	2.45 (1.21 ~ 4.95)
DCE-US Rad-score	1.42	0.42	3.41	<0.001	4.15 (1.83 ~ 9.39)

OR, Odds Ratio; CI, Confidence Interval.

The prediction performance data of each model are presented in **Table 4**, and the corresponding ROC curves are shown in **Figure 2**. In the training cohort, the AUC of the combined model was 0.936 (95%CI: 0.895-0.978), which was better than the clinical ultrasound model (AUC = 0.825, 95%CI: 0.751-0.900) and the radiomics model (AUC = 0.876, 95%CI: 0.818-0.935). In the validation cohort, the AUCs of the three models were 0.916 (95% CI: 0.837–0.995), 0.760 (95% CI: 0.640–0.881), and 0.800 (95% CI: 0.677–0.924), respectively. The sensitivity and specificity of the combined model in the training cohort were 88.3% and 91.8%, respectively. The sensitivities and specificities in the validation cohort reached 80.8% and 87.5%, respectively (**Table 4**). The Delong test showed that the resolution was significantly higher than that of the other two models (both P<0.05) in both the training and validation cohorts (**Table 5**). A nomogram was drawn based on the combined model with optimal efficiency (**Figure 3**). Detailed data of the two cases are provided in the **Supplementary Material 3**.

**Table 4:** Diagnostic performance of each model in the training cohort and validation cohort.

s		AUC (95%CI)	Accuracy (95% CI)	Sensitivity (95% CI)	Specificity (95% CI)	PPV (95% CI).	NPV (95% CI).
Training cohort (n=133)	Model1	0.825 (0.751-0.900)	0.820 (0.744-0.881)	0.733 (0.621-0.845)	0.890 (0.819-0.962)	0.846 (0.748-0.944)	0.802 (0.716-0.889)
	Model2	0.876 (0.818-0.935)	0.827 (0.752-0.887)	0.750 (0.640-0.860)	0.890 (0.819-0.962)	0.849 (0.753-0.945)	0.813 (0.727-0.898)
	Model3	0.936 (0.895-0.978)	0.902 (0.839-0.947)	0.883 (0.802-0.965)	0.918 0.855-0.981)	0.898 (0.821-0.975)	0.905 (0.839-0.972)
Validation cohort (n=58)	Model1	0.760 (0.640-0.881)	0.655 (0.519-0.775)	0.577 (0.387-0.767)	0.719( 0.563-0.875)	0.625 (0.431-0.819)	0.676 (0.519-0.834)
	Model2	0.800 (0.677-0.924)	0.776 (0.647-0.875)	0.615 (0.428-0.802)	0.906 (0.805-1.000)	0.842 (0.678-1.000)	0.744 (0.607-0.881)
	Model3	0.916 (0.837-0.995)	0.845 (0.726-0.927)	0.808 (0.656-0.959)	0.875 (0.760-0.990)	0.840 (0.696-0.984)	0.848 (0.726-0.971)

**Figure 2:**
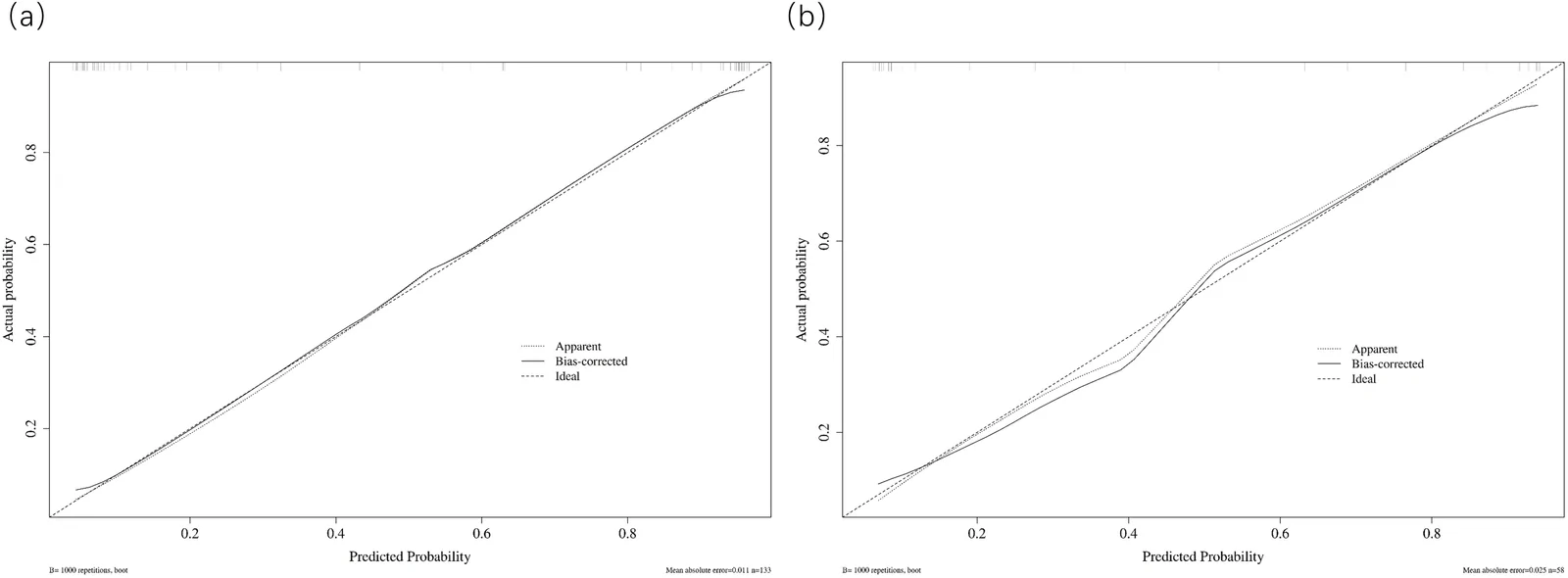
Calibration curves of the combined model in the training **(A)** and validation **(B)** cohorts.

**Table 5:** AUC comparison between different prediction models in the training cohort and validation cohort.

Model	Training cohort (n = 133)		Validation cohort (n = 58)	
	AUC	*P*	AUC	*P*
Clinical-ultrasound model VS Radiomics model	0.825 VS 0.876	*P* = 0.3010	0.760 VS 0.800	*P* = 0.2000
Clinical-ultrasound model VS Combined model	0.825 VS 0.936	*P* = 0.0003	0.760VS 0.916	*P* = 0.0065
Radiomics model VS Combined model	0.876 VS 0.936	*P* = 0.0211	0.800 VS 0.916	*P* = 0.0444

**Figure 3:**
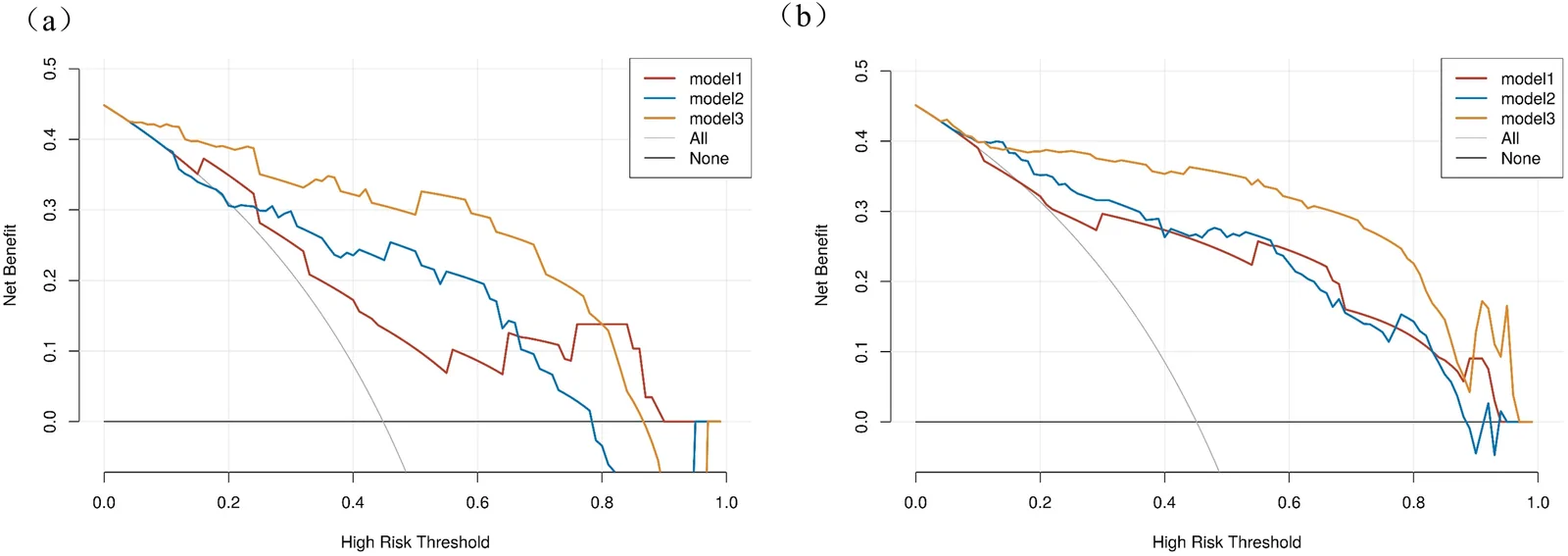
DCA curves of three models in the training **(a)** and validation **(b)** cohort.

The calibration curves showed that the prediction probability of the nomogram was in good agreement with the actual incidence of CLNM in the training cohort and validation cohort (**Figure 4**).The Hosmer–Lemeshow test showed that the model fit effect was good (training cohort P = 0.7904;validation cohort P = 0.2498).Decision curve analysis showed that the clinical net benefit of the combined nomogram was higher than that of the clinical ultrasound model and the radiomics model (**Figure 3**).SHAP analysis showed that DCE-US radiomics score and TR-Ab were important predictors (**Figure 5**).NRI and IDI analyses further confirmed that the predictive ability of the combined model was significantly better than those of the radiomics and clinical ultrasound models (**Table 6**). The clinical ultrasound and radiomics models were used as the old models, and the combined model was analyzed as the new model. After adding radiomics scores, the combined model significantly improved the predictive reclassification ability of CLNM risk compared with the clinical ultrasound model. The results show that the IDI in the training cohort is 26.4% (95% CI: 18.3%-34.5%, P<0.001), and the IDI in the validation cohort was 32.6% (95% CI:22.0%-43.3%, P<0.001).Similarly, after incorporating clinical ultrasound indicators, the combined model also significantly improved the reclassification efficiency of CLNM risk compared with the radiomics model. The results showed that the IDI in the training cohort was 20.7% (95% CI:12.7%-28.6%, P <0.001), and the IDI in the validation cohort was 28.4% (95% CI:14.5%-42.4%, P<0.001). To further evaluate the clinical application value of the combined model in real-world clinical decision-making, validation in a restricted population was performed in this study. Among the 58 patients in the validation cohort, 51 patients met the criteria for the central lymph node metastasis subgroup, including 19 metastasis-positive cases and 32 negative cases. Seven patients were excluded due to concurrent lateral cervical lymph node metastasis. Prediction was performed using the combined model. The results demonstrated an AUC of 0.875 (95% CI: 0.780–0.970), with a sensitivity of 68.4%, specificity of 84.4%, and accuracy of 78.4% (**Table 7**). The calibration curve indicated excellent goodness of fit (Hosmer–Lemeshow test, P = 0.3227). Decision curve analysis (DCA) revealed that the model provided a favorable net clinical benefit across a wide range of threshold probabilities (**Figure 3**).

**Figure 4:**
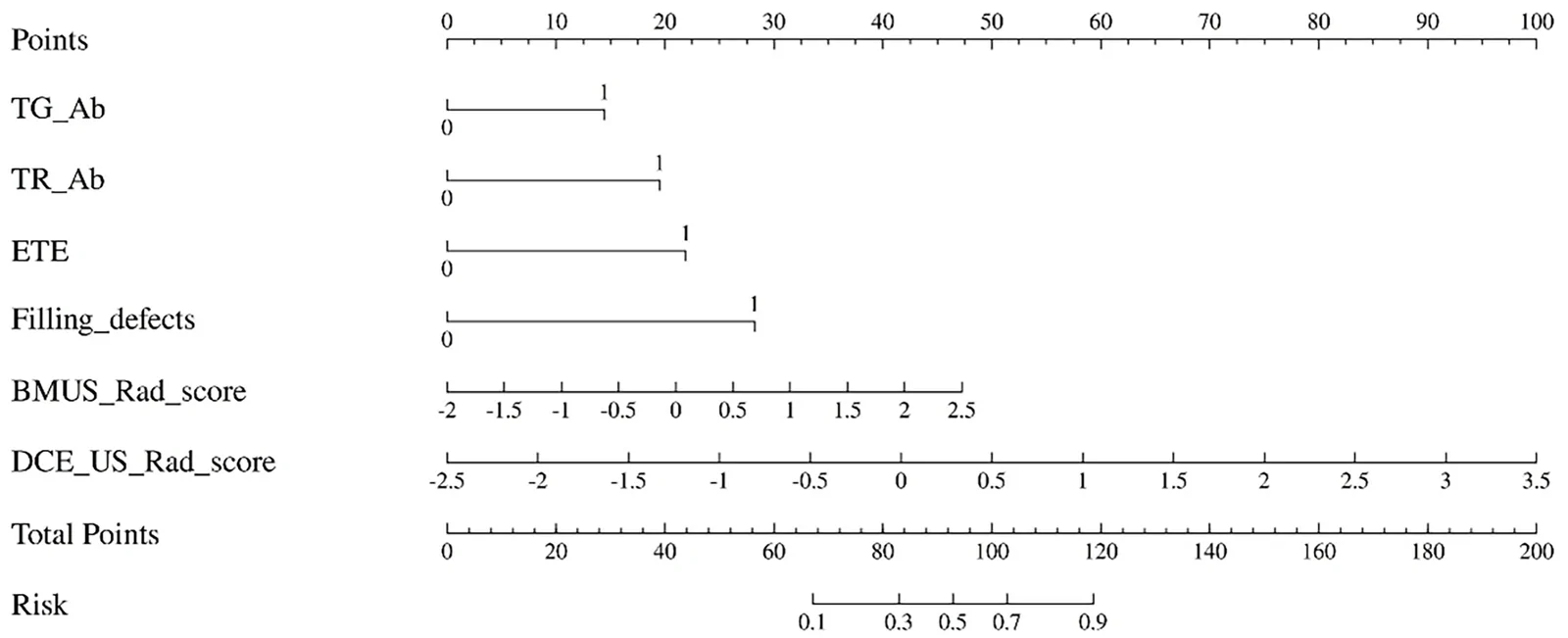
Nomogram of the combined model.

**Figure 5:**
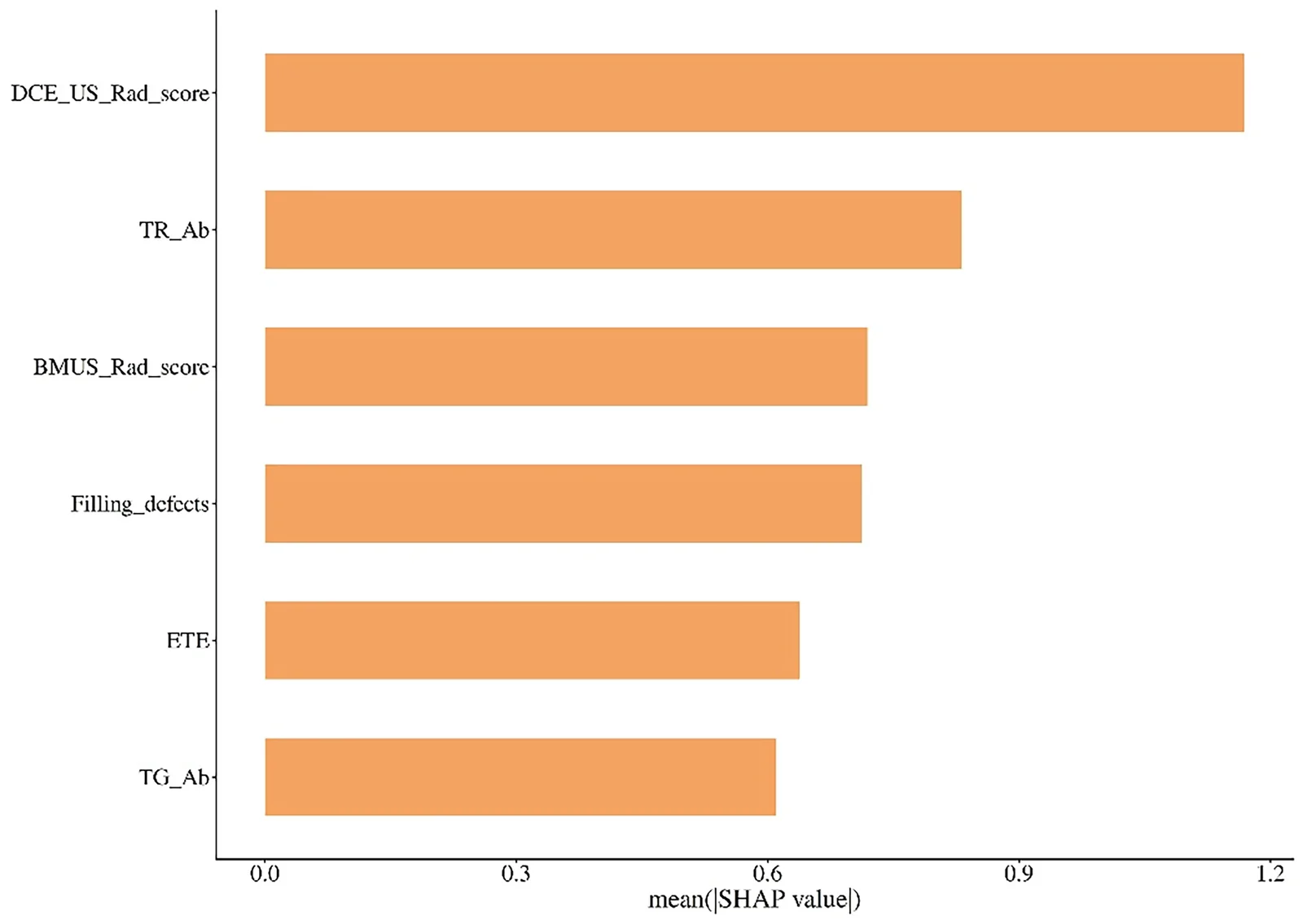
The importance distribution of SHAP values of each feature of the combined model.

**Table 6:** Clinical - comparison of predictive improvement between ultrasound, radiomics, and combined models.

Model	Training cohort (n = 133)		Validation cohort (n = 58)	
	Continuous NRI (95% CI)	IDI (95% CI)	Continuous NRI (95% CI)	IDI (95% CI)
Clinical-ultrasound model VS Combined model	126.5% (100.0%-153.1%)	26.4% (18.3%-34.5%)	149.0% (114.4%-183.7%)	32.6% (22.0%-43.3%)
	*P* < 0.001	*P* < 0.001	*P* < 0.001	*P* < 0.001
Radiomics model VS Combined model	129.2% (103.1%-155.4%)	20.7% (12.7%-28.6%)	102.4% (57.9%-146.9%)	28.4% (14.5%-42.4%)
	*P* < 0.001	*P* < 0.001	*P* < 0.001	*P* < 0.001

**Table 7:** Diagnostic performance of the combined model for central and overall cervical lymph node metastasis.

Results	Central lymph node metastasis	Cervical lymph node metastasis
Positive cases/Total cases	19/51	26/58
AUC (95% CI)	0.875 (0.780-0.970)	0.916 (0.837-0.995)
Accuracy (95% CI)	0.784 (0.647-0.887)	0.845 (0.726-0.927)
Sensitivity (95% CI)	0.684 (0.475-0.893)	0.808 (0.656-0.959)
Specificity (95% CI)	0.844 (0.718-0.970)	0.875 (0.760-0.990)
PPV (95% CI)	0.722 (0.515-0.929)	0.840 (0.696-0.984)
NPV (95% CI)	0.818 (0.687-0.950)	0.848 (0.726-0.971)

**Figure 6:**
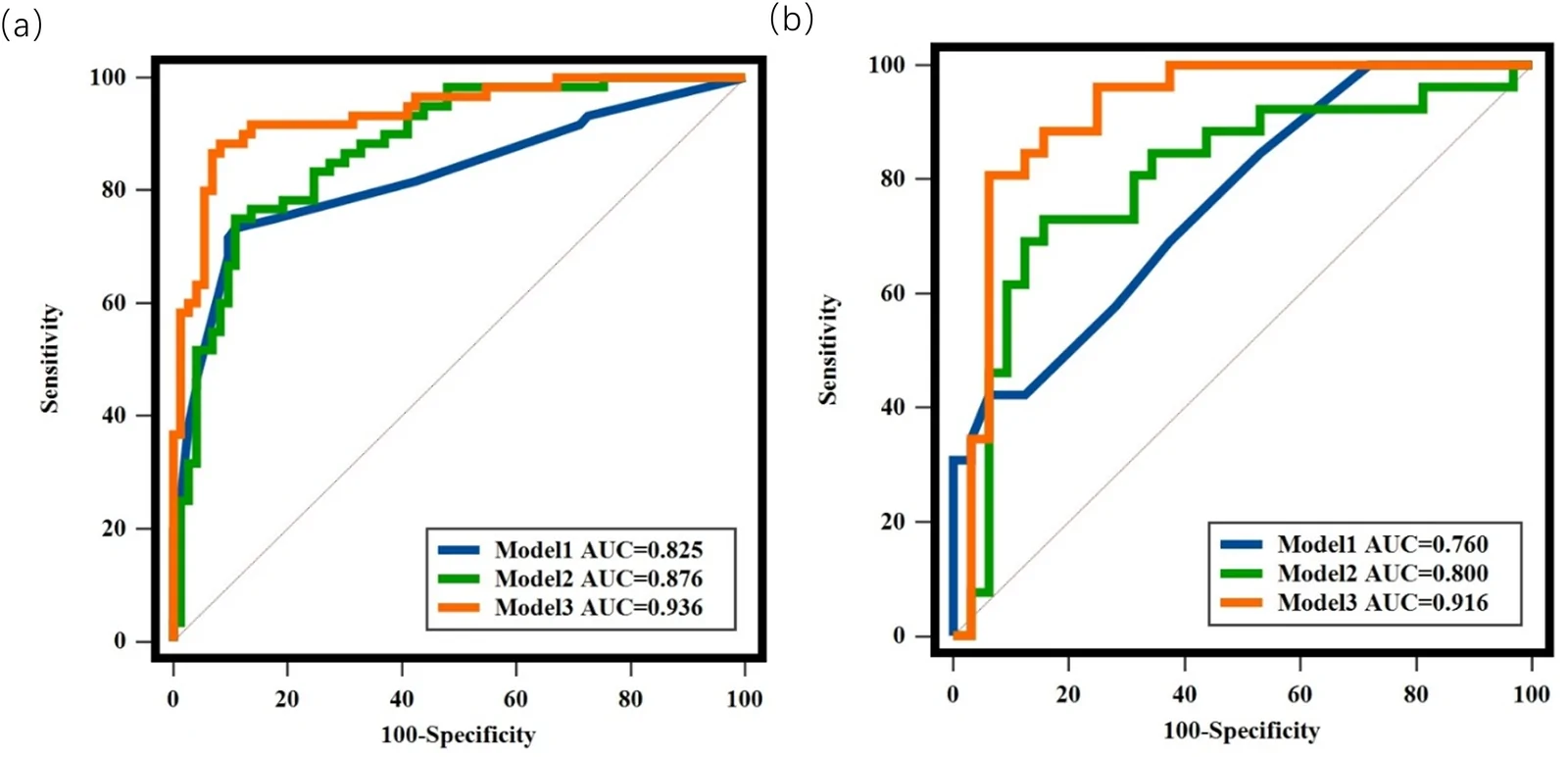
Receiver operating characteristic (ROC) curves of different models in the training **(A)** and validation **(B)** cohorts.

**Figure 7:**
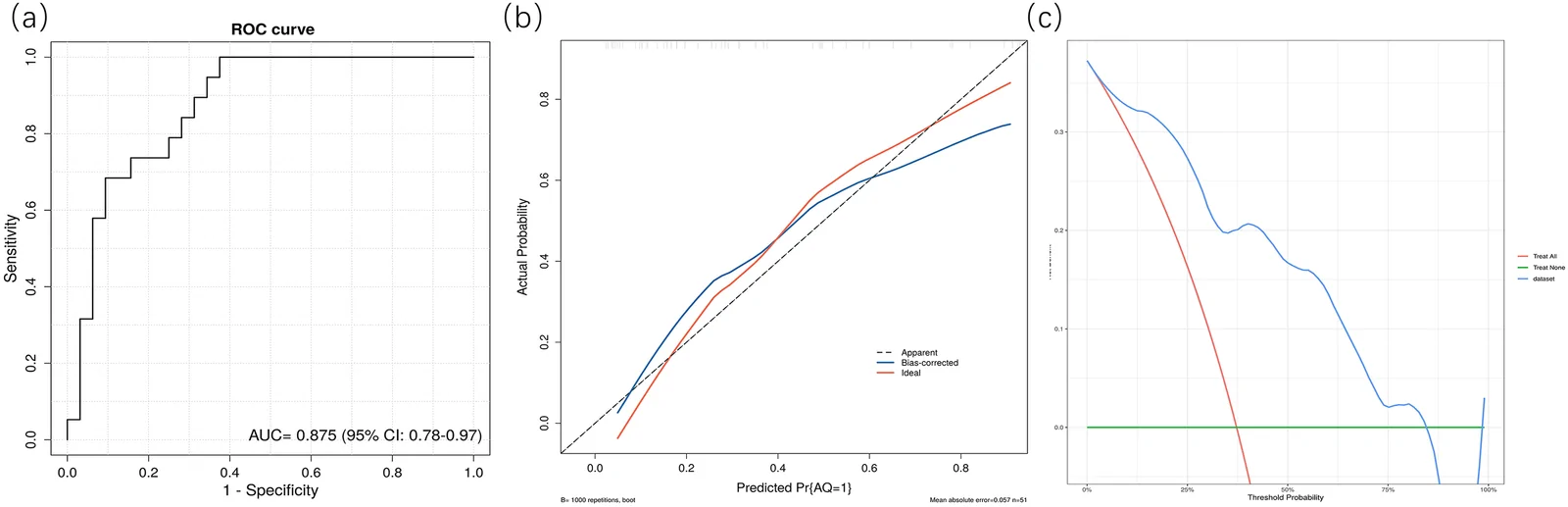
Model evaluation of predicting subgroups of central lymph node metastasis **(A)** ROC curve of the combined model **(B)** calibration curve **(C)** decision curve analysis.

Compared with its predictive performance for cervical lymph node metastasis (AUC = 0.916), the model maintained a relatively high diagnostic ability in the isolated central lymph node metastasis subgroup (AUC = 0.875), suggesting its promising predictive potential for early central lymph node metastasis.

## 3 Discussion

Preoperative accurate assessment of CLNM status is crucial for the formulation of surgical plans for PTC patients, which is directly related to the selection of lymph node dissection (10).At present, ultrasound is the preferred imaging method for evaluating cervical lymph nodes, but its interpretation is highly subjective due to operator experience (11);It is mainly based on the visible ultrasound characteristics such as lymph node size, lymphatic gate morphology, internal echo, and blood flow signal, which is heterogeneous among patients, and it is difficult to provide stable quantitative indicators (12), especially in combination with HT, abnormally enlarged lymph nodes further increase the difficulty of diagnosis (13).CEUS can display blood flow perfusion patterns, which has unique advantages in blood flow assessment; however, the image features that can be recognized by the human eye are limited, and subjective interpretation is still limited by experience, thus limiting its application in the accurate prediction of CLNM. Radiomics extracts quantitative features from medical images at high throughput (6), revealing the spatial heterogeneity and biological characteristics of tumors, and making up for the limitations of traditional ultrasound subjective interpretation and feature extraction (14).DCE-US not only provides morphological information, but also its perfusion parameters can reflect the microvascular density, permeability, and blood flow pattern of the lesion, which are closely related to tumor aggressiveness and metastatic potential (15), providing more specific quantitative features for radiomics analysis (16).However, most current studies are based on BMUS radiomics, and few involve DCE-US radiomics, providing an entry point for this study.

At present, multimodal ultrasound radiomics models have shown excellent application efficiency in the prediction and differential diagnosis of thyroid diseases (17–19).Ren et al. integrated the radiomics characteristics of BMUS and CEUS and constructed a dual modal ultrasound radiomics nomogram based on clinical and ultrasound indicators ACR TI-RADS 4 and 5 thyroid nodules were benign and malignant, the AUCs of the training cohort and the validation cohort reached 0.873 and 0.851, respectively, which could effectively reduce unnecessary fine-needle aspiration biopsy operations in clinical practice. Islam et al.’s deep learning models based on BMUS, color Doppler and shear wave elastography also achieved an AUC of 0.97 in the test cohort, which is significantly better than the single mode (18). Beyond the well-established roles of conventional ultrasound and elastography in thyroid nodule risk stratification (20, 21), accurate preoperative prediction of cervical lymph node metastasis remains challenging, particularly in patients with HT-PTC, where reactive lymphadenopathy and parenchymal heterogeneity complicate image interpretation. While strain elastography has demonstrated value in assessing nodule stiffness and predicting malignancy in TIRADS 4 nodules (21), and Tsukuba elasticity scores have been associated with Bethesda undetermined cytology (20), these modalities primarily reflect tissue stiffness rather than the microvascular functional status of the primary tumor, which is critical for metastatic potential. This limitation underscores the need for complementary functional imaging approaches, such as DCE-US parametric imaging, to capture perfusion heterogeneity—a dimension not accessible by conventional grayscale ultrasound or elastography alone. A prospective multicenter study by Yao et al. confirmed that the combined model integrating elastography features and radiomics features had an AUC of 0.863 in the test cohort, and could effectively improve the diagnosis level of junior physicians (19).These studies show that multimodal radiomics models can significantly improve the accuracy and objectivity of diagnosis by integrating multidimensional ultrasound information, such as morphology, hemodynamics, and stiffness.

Based on existing research, this study focused on patients with HT and PTC and constructed a combined prediction model integrating clinical, ultrasound image, and radiomics features to assess CLNM risk. Predictive indicators of the multimodal ultrasound radiomics model included TG-Ab, TR-Ab, ETE, deletion of enhancement, and BMUS and DCE-US radiomics scores. Results showed that CLNM prediction efficiency of the combined model was significantly better than that of clinical ultrasound and radiomics models alone. DCA showed that the combined model had wider curve coverage and higher clinical net returns across the entire threshold interval, suggesting good clinical application potential. SHAP analysis further showed that the DCE-US radiomics score and ETE were the two most important predictors, with the DCE-US radiomics score contributing most, while the BMUS radiomics score also contributed. In addition, NRI and IDI increased significantly after adding BMUS and CEUS radiomics features to the clinical ultrasound model, indicating that both rad-scores are high-value markers for predicting CLNM in patients with HT and PTC. The radiomics nomogram effectively solves clinicians’ difficulty in predicting CLNM.

The enhanced robustness of the nomogram developed in this study can be attributed to the integration of dual-modal imagistic features from both BMUS and DCE-US. In comparison, Jin et al. constructed a nomogram for predicting cervical lymph node metastasis using only clinical data and single-modal ultrasound radiomics features. Their optimal model achieved an AUC of 0.808 in the test cohort (22). The limitation of single-modal approaches is further illustrated by the work of Vivian Y. et al. (23), who extracted radiomics features—including texture, wavelet, and first-order statistics—from BMUS images of primary tumors. In the validation cohort, the radiomics model achieved an AUC of 0.710, which was considerably lower than that of the combined model established in the present study (AUC = 0.916). Building on these previous studies, our study further advances current research by integrating dual-modal DCE-US radiomics features with conventional clinical and BMUS characteristics. This integrative strategy not only captures standard morphological features of the lesion but also incorporates multidimensional information, including contrast-enhanced ultrasound kinetic patterns and clinical antibody markers. The incorporation of such heterogeneous data is crucial to the marked improvement in the predictive performance of our model.

To clarify the importance of radiomic features in predicting CLNM, this study found that radiomics scores encompass multiple features, predominantly wavelet features. These describe the spatial distribution of lesion voxels, and wavelet transformation features offer advantages in characterizing tumor biological behavior. This confirms that PTC exhibits high vascular heterogeneity, predisposing it to aggressive behavior and reflecting the grayscale and textural heterogeneity of HT and PTC lesions. Wavelet transform can extract potential image information at a multiscale level, thereby amplifying heterogeneous differences within lesions (24), and enhancing the model’s prediction efficiency (25)This also indicates that a radiomics model combining ultrasonography and wavelet features holds greater application value in evaluating CLNM. Wang et al.’s study confirmed that wavelet transform is an effective ultrasound image filtering method for predicting cervical lymph node metastasis on the PTC side, with its high-pass filtering effectively enhancing the diagonal edges and details of the image, thus providing diagnostic edge and morphological characteristics (26); Wei et al. demonstrated that the AUC in thyroid, breast, and kidney ultrasound datacohorts reached 0.805-0.915, suggesting its potential as a novel imaging biomarker for ultrasound histological diagnosis (27).Jiang et al.’s univariate analysis results showed that BMUS and contrast-enhanced ultrasound radiomics scores were correlated with thyroid neck lymph node metastasis, while only contrast-enhanced ultrasound radiomics scores were included in the multivariate stepwise regression analysis, with wavelet features accounting for 80% (28). These radiomics features, which are challenging to identify with the naked eye, are anticipated to serve as noninvasive preoperative predictors for assessing CLNM status in patients with PTC.

Several limitations of this study should be acknowledged when interpreting the findings. First, this was a single-center retrospective study that enrolled 191 patients from a single institution, and no external validation dataset from an independent center was available. Although our radiomic signatures demonstrated promising performance in the internal test set, their generalizability to broader populations warrants further validation through multicenter, large-sample prospective studies. Second, ROI delineation was performed manually, which is inherently operator-dependent and may introduce subjective bias. Although all ultrasound features were evaluated by consensus among experienced reviewers, inter-observer agreement was not formally quantified using Kappa statistics, which may affect the reproducibility of qualitative feature assessments in other settings. Additionally, although CEUS acquisition was performed using a standardized protocol, inter-operator variability was not systematically assessed, which may affect the reproducibility of feature extraction. Third, this study employed a sequential feature selection pipeline consisting of the Mann-Whitney U test, RFE, and LASSO regression. While LASSO was selected for its advantages in feature selection, parsimony, and clinical interpretability, we acknowledge that other machine learning algorithms—such as Random Forest or XG Boost—might yield different performance profiles. Future studies with larger cohorts are warranted to compare multiple algorithms and further validate our findings. The feature selection strategy and modelling approach may also affect model stability and predictive efficiency; therefore, subsequent research should explore optimized selection procedures and incorporate artificial intelligence techniques, such as deep learning, to enhance predictive performance. Fourth, we did not stratify patients by HT severity based on antibody titers or ultrasound-graded heterogeneity. Given that background parenchymal heterogeneity in HT may affect the robustness of BMUS radiomic features, the potential confounding effect of HT severity on model performance remains unclear and merits further investigation in larger, stratified cohorts. Finally, the biological mechanisms underlying the identified radiomic features have not been fully elucidated. Follow-up studies should conduct in-depth analyses in conjunction with multi-omics data—such as genomics and pathomics—to clarify their potential roles in thyroid cancer initiation, progression, and postoperative repair.

## 4 Conclusion

In this study, a multimodal combined model was developed by integrating BMUS and DCE-US radiomics features with clinical indicators, significantly enhancing the preoperative prediction efficiency of CLNM in patients with HT and PTC. Presented as a nomogram, this model offers an intuitive reference for clinical decision-making. This research broadens the application scope of multimodal ultrasound imaging in the precise assessment of thyroid cancer and introduces new approaches for integrating multidimensional information to construct a personalized prediction model.

## Data Availability

The datasets presented in this article are not readily available because The original contributions presented in the study are included in the article/**Supplementary Material**. Further inquiries can be directed to the corresponding author. Requests to access the datasets should be directed to fldong@suda.edu.cn.

## Glossary

AUC: Area under the curve
BMUS: B-mode ultrasound
CEUS: Contrast-enhanced ultrasound
CI: Confidence interval
CLNM: Cervical lymph node metastasis
DCA: Decision curve analysis
DCE-US: Dynamic contrast-enhanced ultrasound
DICOM: Digital imaging and communications in medicine
ETE: Extrathyroidal extension
GLCM: Gray level cooccurrence matrix
GLDM: Gray level dependence matrix
GLRLM: Gray level run-length matrix
GLSZM: Gray level size zone matrix
HT: Hashimoto’s thyroiditis
ICC: Intra- and interclass correlation coefficient
IDI: Integrated discrimination improvement
LASSO: Least absolute shrinkage and selection operator
NRI: Net reclassification improvement
PE: Peak enhancement
PI: Peak intensity
PTC: Papillary thyroid carcinoma
Rad-score: Radiomics score
ROC: Receiver operating characteristic
ROI: Region of interest
RT: Rise time
TG-Ab: Thyroglobulin antibody
TICs: Time-intensity curves
TNs: Thyroid nodules
TPO-Ab: Thyroid peroxidase antibody
TR-Ab: Thyrotropin receptor antibody

## References

[1] Song E Jeon MJ Park S Kim M Oh HS Song DE . Influence of coexistent Hashimoto's thyroiditis on the extent of cervical lymph node dissection and prognosis in papillary thyroid carcinoma. Clin Endocrinol (Oxf). (2018) 88:123–8. doi: 10.1111/cen.13475 28906015

[2] Wang MF Xia H Cai J . The impact of coexisting Hashimoto's thyroiditis on the feasibility of endoscopic thyroidectomy in papillary thyroid carcinoma. Heliyon. (2024) 10:e26793. doi: 10.1016/j.heliyon.2024.e26793 38434093 PMC10906291

[3] Gao J Liu Y Zheng L Wang X Wang Y Zhou T . Diagnostic performance of contrast-enhanced ultrasound vs. conventional ultrasound for lymph node metastasis in patients with thyroid cancer: A meta-analysis. Oncol Lett. (2025) 30:407. doi: 10.3892/ol.2025.15153 40613033 PMC12217433

[4] Huang Y Wang Y Liu L Zhu L Qiu Y Zuo D . VueBox® perfusion analysis of dynamic contrast enhanced ultrasound provides added value in the diagnosis of small thyroid nodules. Clin Hemorheol Microcirc. (2023) 83:409–20. doi: 10.3233/ch-221681 36683500

[5] Zhao HN Yin H Li MH Zhang HQ He YS Luo HH . Contrast-enhanced ultrasound image sequences based on radiomics analysis for diagnosis of metastatic cervical lymph nodes from thyroid cancer. Gland Surg. (2024) 13:1437–47. doi: 10.21037/gs-24-98 39282044 PMC11399000

[6] Liu C Yang S Xue T Zhang Q Zhang Y Zhao Y . The application of a clinical-multimodal ultrasound radiomics model for predicting cervical lymph node metastasis of thyroid papillary carcinoma. Front Oncol. (2024) 14:1507953. doi: 10.3389/fonc.2024.1507953 39896179 PMC11782237

[7] Wei T Wei W Ma Q Shen Z Lu K Zhu X . Development of a clinical-radiomics nomogram that used contrast-enhanced ultrasound images to anticipate the occurrence of preoperative cervical lymph node metastasis in papillary thyroid carcinoma patients. Int J Gen Med. (2023) 16:3921–32. doi: 10.2147/ijgm.s424880 37662506 PMC10474867

[8] Su B Li L Liu Y Liu H Zhan J Chai Q . Quantitative parameters of contrast-enhanced ultrasound effectively promote the prediction of cervical lymph node metastasis in papillary thyroid carcinoma. Drug Discov Ther. (2024) 18:44–53. doi: 10.5582/ddt.2023.01095 38355122

[9] Zhan J Li F Ma J Li X Liu L Wu R . Qualitative and quantitative prediction of central cervical lymph node metastases by utilizing contrast-enhanced ultrasound in papillary thyroid carcinoma with a special focus on peak intensity ratio. Ultrasound Med Biol. (2026) 52:506–13. doi: 10.1016/j.ultrasmedbio.2025.09.023 41407611

[10] Fu J Liu J Wang Z Qian L . Predictive values of clinical features and multimodal ultrasound for central lymph node metastases in papillary thyroid carcinoma. Diagnostics (Basel). (2024) 14 (16), 1770. doi: 10.3390/diagnostics14161770 39202260 PMC11353660

[11] de Carlos J Garcia J Basterra FJ Pineda JJ Ollero MD Toni M . Interobserver variability in thyroid ultrasound. Endocrine. (2024) 85:730–6. doi: 10.1007/s12020-024-03731-5 38372907

[12] Wang X Qi Y Zhang X Liu F Li J . Ultrasound-based artificial intelligence for predicting cervical lymph node metastasis in papillary thyroid cancer: a systematic review and meta-analysis. Front Endocrinol (Lausanne). (2025) 16:1570811. doi: 10.3389/fendo.2025.1570811 40556829 PMC12185295

[13] Wang L Zhang L Wang D Chen J Su W Sun L . Predicting central cervical lymph node metastasis in papillary thyroid carcinoma with Hashimoto's thyroiditis: a practical nomogram based on retrospective study. PeerJ. (2024) 12:e17108. doi: 10.7717/peerj.17108 38650652 PMC11034492

[14] Wildeboer RR Mannaerts CK van Sloun RJG Budäus L Tilki D Wijkstra H . Automated multiparametric localization of prostate cancer based on B-mode, shear-wave elastography, and contrast-enhanced ultrasound radiomics. Eur Radiol. (2020) 30:806–15. doi: 10.1007/s00330-019-06436-w 31602512 PMC6957554

[15] Elie N Kaliski A Péronneau P Opolon P Roche A Lassau N . Methodology for quantifying interactions between perfusion evaluated by DCE-US and hypoxia throughout tumor growth. Ultrasound Med Biol. (2007) 33:549–60. doi: 10.1016/j.ultrasmedbio.2006.09.011 17350158

[16] Naccache R Belkouchi Y Lawrance L Benatsou B Hadchiti J Cournede PH . Prediction of early response to immunotherapy: DCE-US as a new biomarker. Cancers (Basel). (2022) 14 (5), 1337. doi: 10.3390/cancers14051337 35267645 PMC8909556

[17] Ren JY Lv WZ Wang L Zhang W Ma YY Huang YZ . Dual-modal radiomics nomogram based on contrast-enhanced ultrasound to improve differential diagnostic accuracy and reduce unnecessary biopsy rate in ACR TI-RADS 4-5 thyroid nodules. Cancer Imaging. (2024) 24:17. doi: 10.1186/s40644-024-00661-3 38263209 PMC10807093

[18] Saini M Parvar TA Velarde M Larson NB Fatemi M Alizad A . Deep learning-based classification of thyroid nodules using uncertainty-aware multi-modal ultrasound imaging. Sci Rep. (2026) 16:4938. doi: 10.1038/s41598-026-35965-w 41526676 PMC12873121

[19] Cui S Liu Q Wang H Li H Li W Li C . The value of a combined model based on ultra-radiomics and multi-modal ultrasound in the benign-malignant differentiation of C-TIRADS 4A thyroid nodules: a prospective multicenter study. Front Oncol. (2025) 15:1543020. doi: 10.3389/fonc.2025.1543020 40406245 PMC12095005

[20] Sengul D Sengul I . Association between Tsukuba elasticity scores 4 and 5 on elastography and Bethesda undetermined cytology on US-guided FNA with 27-G needle, verified by histopathology: a cut-off point of 20 mm of diameter designated for thyroid nodules. J Buon. (2019) 24:382–90. 30941995

[21] Sengul D Sengul I Egrioglu E Ozturk T Aydin I Kesicioglu T . Can cut-off points of 10 and 15 mm of thyroid nodule predict Malignancy on the basis of three diagnostic tools: i) strain elastography, ii) the Bethesda System for Reporting Thyroid Cytology with 27-gauge fine-needle, and iii) histopathology? J Buon. (2020) 25:1122–9. doi: 10.3389/fendo.2022.993564 32521915

[22] Jin P Chen J Dong Y Zhang C Chen Y Zhang C . Ultrasound-based radiomics nomogram combined with clinical features for the prediction of central lymph node metastasis in papillary thyroid carcinoma patients with Hashimoto's thyroiditis. Front Endocrinol (Lausanne). (2022) 13:993564. doi: 10.3389/fendo.2022.993564 36060946 PMC9439618

[23] Park VY Lee E Lee HS Kim HJ Yoon J Son J . Combining radiomics with ultrasound-based risk stratification systems for thyroid nodules: an approach for improving performance. Eur Radiol. (2021) 31:2405–13. doi: 10.1007/s00330-020-07365-9 33034748

[24] Abbasian Ardakani A Reiazi R Mohammadi A . A clinical decision support system using ultrasound textures and radiologic features to distinguish metastasis from tumor-free cervical lymph nodes in patients with papillary thyroid carcinoma. J Ultrasound Med. (2018) 37:2527–35. doi: 10.1002/jum.14610 29603330

[25] Chung HJ Han K Lee E Yoon JH Park VY Lee M . Radiomics analysis of gray-scale ultrasonographic images of papillary thyroid carcinoma > 1 cm: potential biomarker for the prediction of lymph node metastasis. J Korean Soc Radiol. (2023) 84:185–96. doi: 10.3348/jksr.2021.0155 36818698 PMC9935950

[26] Ding X Liu Y Zhao J Wang R Li C Luo Q . A novel wavelet-transform-based convolution classification network for cervical lymph node metastasis of papillary thyroid carcinoma in ultrasound images. Comput Med Imaging Graph. (2023) 109:102298. doi: 10.1016/j.compmedimag.2023.102298 37769402

[27] Wei Q Zeng S Jiang Z Xu F Shi S Wen W . Topological representation based on wavelet transform as a novel imaging biomarker for tumor diagnosis in ultrasound images: a comprehensive study. Comput Methods Programs BioMed. (2025) 269:108859. doi: 10.1016/j.cmpb.2025.108859 40517521

[28] Jiang L Zhang Z Guo S Zhao Y Zhou P . Clinical-radiomics nomogram based on contrast-enhanced ultrasound for preoperative prediction of cervical lymph node metastasis in papillary thyroid carcinoma. Cancers (Basel). (2023) 15. doi: 10.3390/cancers15051613 36900404 PMC10001290

