# Supplementary material for: Multimodal radiomics based on dynamic contrast-enhanced ultrasound for predicting cervical lymph node metastasis in papillary thyroid carcinoma with Hashimoto’s thyroiditis
Source: Front Oncol. 2026 Oct 14;16:1840185. doi: 10.3389/fonc.2026.1840185 (PMC13713338; doi:10.3389/fonc.2026.1840185)
Supplement: Supplementary file 1 [file SupplementaryFile1.docx]

| Section | Item Number | CLAIM Item | Location in Manuscript | Reporting Status |
| --- | --- | --- | --- | --- |
| **Title & Abstract** | 1 | Title clearly identifies the study as AI/radiomics research | Title page | ✓ |
|  | 2 | Abstract summarizes purpose, methods, and key findings | Abstract | ✓ |
| **Introduction** | 3 | Background and clinical problem clearly stated | Introduction, Para 1–2 | ✓ |
|  | 4 | Study objective and hypothesis explicitly stated | Introduction, Para 3 | ✓ |
| **Methods** | 5 | Study design (retrospective/prospective) described | Methods 1.1 | ✓ |
|  | 6 | Inclusion/exclusion criteria defined | Methods 1.1 | ✓ |
|  | 7 | Data collection period and site described | Methods 1.1 | ✓ |
|  | 8 | Sample size and rationale provided | Methods 1.1 | ✓ |
|  | 9 | Data preprocessing methods described | Methods 1.3.2 | ✓ |
|  | 10 | Data splitting method (training/validation) described | Methods 1.1 | ✓ |
|  | 11 | ROI annotation method and operator qualifications described | Methods 1.3.1 | ✓ |
|  | 12 | Reproducibility assessment (ICC) described | Methods 1.3.2 | ✓ |
|  | 13 | Radiomics feature types specified | Methods 1.3.2 | ✓ |
|  | 14 | Feature extraction software and version specified | Methods 1.3.2 | ✓ |
|  | 15 | Feature selection method described | Methods 1.3.3 | ✓ |
|  | 16 | Modeling algorithm specified | Methods 1.3.3 | ✓ |
|  | 17 | Hyperparameter tuning method described | Methods 1.3.3 | ✓ |
|  | 18 | Model evaluation metrics defined | Methods 1.4 | ✓ |
|  | 19 | Statistical tests described | Methods 1.5 | ✓ |
|  | 20 | Multiple comparison correction reported | Methods 1.5 | ✓ |
| **Results** | 21 | Patient baseline characteristics reported | Results 2.1, Table 1 | ✓ |
|  | 22 | Model performance metrics reported with CIs | Results 2.3, Table 4 | ✓ |
|  | 23 | Calibration results reported | Results 2.3, Fig 4 | ✓ |
|  | 24 | DCA results reported | Results 2.3, Fig 5 | ✓ |
|  | 25 | NRI/IDI results reported with CIs | Results 2.3, Table 6 | ✓ |
|  | 26 | SHAP analysis results reported | Results 2.3, Fig 6 | ✓ |
| **Discussion** | 27–41 | Interpretation and limitations discussed | Discussion, Para 1–6 | ✓ |
