# Supplementary material for: Multimodal radiomics based on dynamic contrast-enhanced ultrasound for predicting cervical lymph node metastasis in papillary thyroid carcinoma with Hashimoto’s thyroiditis
Source: Front Oncol. 2026 Oct 14;16:1840185. doi: 10.3389/fonc.2026.1840185 (PMC13713338; doi:10.3389/fonc.2026.1840185)
Supplement: Supplementary file 2 [file SupplementaryFile2.docx]

Case 1: Low‑Risk Case

A middle‑aged male patient presented with preoperative TG‑Ab > 60 IU/mL, while TR‑Ab was within normal range (≤ 1.75 IU/L). No extrathyroidal extension (ETE) or filling defects were detected on B‑mode ultrasound and contrast‑enhanced ultrasound. The radiomics scores (Rad‑scores) derived from BMUS and DCE‑US were 1.01749 and –0.43096, respectively.

Using the nomogram, the individual point contributions were as follows: TG‑Ab positivity contributed 51 points; BMUS Rad‑score contributed 38.0 points; DCE‑US Rad‑score contributed –25.4 points; the other clinical factors (TR‑Ab, ETE, and filling defects) were all negative, thus contributing 0 points each. The total points amounted to 63.6, corresponding to a predicted probability of CLNM of approximately 26.0%. This case was classified as low risk, suggesting that a conservative follow‑up strategy could be considered, avoiding unnecessary prophylactic lymph node dissection.

Case 2: High‑Risk Case

A middle‑aged female patient also had TG‑Ab > 60 IU/mL, with normal TR‑Ab, and no ETE or filling defects. Her BMUS and DCE‑US Rad‑scores were 0.71359 and 0.91938, respectively. The nomogram scoring showed: TG‑Ab positivity added 51 points; BMUS Rad‑score added 26.6 points; DCE‑US Rad‑score added 54.2 points; all other factors (TR‑Ab, ETE, filling defects) were negative and contributed 0 points. The total points reached 131.8, corresponding to a predicted CLNM probability of 64.5%. This case was classified as high risk, and we recommend a thorough preoperative ultrasound evaluation of cervical lymph nodes, with a tendency toward prophylactic lymph node dissection.
